# The Effects of Human–Horse Interactions on Oxytocin and Cortisol Levels in Humans and Horses

**DOI:** 10.3390/ani15070905

**Published:** 2025-03-21

**Authors:** Youngwook Jung, Minjung Yoon

**Affiliations:** 1 Department of Animal Science and Biotechnology, Kyungpook National University, Sangju 37224, Republic of Korea; wook02070@gmail.com; 2 Department of Horse, Companion and Wild Animal Science, Kyungpook National University, Sangju 37224, Republic of Korea; 3 Research Institute for Innovative Animal Science, Kyungpook National University, Sangju 37224, Republic of Korea

**Keywords:** human, equine, social interaction, stress response, saliva oxytocin, plasma oxytocin, saliva cortisol, plasma cortisol

## Abstract

Therapeutic programs involving human–horse interactions are gaining popularity as a means of enhancing human well-being. Understanding the physiological responses of both humans and horses during these interactions is essential for evaluating the effectiveness of such programs. This study examined the effects of specific interactive activities on both humans and horses by monitoring changes in oxytocin and cortisol levels. Six participants and six horses took part in the study. The participants engaged in three distinct activities, each lasting 15 min: (1) resting alone without the horse (resting), (2) standing near the horse without physical contact (standing), and (3) gently rubbing the horse’s neck and withers (rubbing). Saliva samples from the participants and blood samples from the horses were collected at three time points for each activity: T0 (before the activity), T1 (at the end of the activity), and T2 (15 min after the activity ended). The results indicated that oxytocin levels significantly increased in horses at T2 following both the standing and rubbing activities, while cortisol levels remained unchanged in both humans and horses across all activities. These findings suggest that human–horse interactions, particularly standing and rubbing, may foster social bonding in horses without eliciting a stress response in either species.

## Simple Summary

Human–horse interaction programs are becoming increasingly popular for improving well-being, yet their biological effects on both humans and horses remain unclear. This study explored whether interactive activities influence oxytocin, a hormone linked to social bonding, and cortisol, a hormone associated with stress, in both humans and horses. The results showed that salivary oxytocin levels in humans did not significantly increase following interactive activities, whereas plasma oxytocin levels in horses significantly increased after standing with humans or being rubbed by them. Cortisol levels remained unchanged in both humans and horses across all activities. These findings suggest that human–horse interactions may strengthen social bonds in horses while not eliciting a stress response in either species. This research provides scientific evidence supporting the benefits of human–horse interaction programs, contributing to the development and advancement of animal-assisted interventions aimed at enhancing both human and animal well-being.

## 1. Introduction

Human–animal interactions (HAIs) have garnered considerable attention owing to their potential benefits for human health and well-being. HAIs have been explored with various species, including dogs [1], cats [2], rabbits [3], guinea pigs [4], and cows [5]. Among these animals, horses have been particularly prominent in HAIs, with typical activities including grooming, leading, and groundwork [6]. Several studies have examined the effects of human–horse interactions (HHIs) on both humans and horses. For instance, Crews investigated electroencephalograph (EEG) synchronization during different interactive activities, such as grooming and petting, between humans and horses. Their results revealed that compared to control activities, EEG synchronization was better during activities like grooming, petting, and standing next to a horse [7]. Baldwin and colleagues also observed that grooming led to synchronized peak frequencies in heart rate variability between humans and horses [8]. These findings suggest that HHIs may influence physiological indicators by promoting social bonding. However, these studies have focused on only a limited set of physiological parameters, highlighting the need for more comprehensive assessments to entirely understand the effects of HHIs. Similarly to brainwave and heart rate indicators, hormone levels are considered reliable physiological indicators, owing to their rapid response to stimulation.

In HAI studies involving animals such as dogs and cats, researchers have frequently measured oxytocin and cortisol levels to assess the effects of HAIs on stress and social bonding. Oxytocin is involved in several physiological functions, such as promoting anti-stress responses [9] and supporting immune activity [10]. In the context of HAIs, oxytocin also plays a crucial role in promoting sociality [11] and fostering familiarity between animals and humans [12,13]. Social interactions between humans and animals, particularly with dogs, are well-established ways to increase oxytocin levels [14,15,16]. While oxytocin is associated with bonding and stress reduction, cortisol is released in response to stress and emotional distress. Cortisol’s rapid response to acute stress makes it a common stress indicator in both humans and domestic animals [17,18]. In HAI studies, cortisol levels are frequently monitored to assess the stress-relieving effects of animal interactions, along with β-endorphin, adrenocorticotropic hormone, corticotropin-releasing hormone, catecholamines, and iodothyronines [19,20]. Studies have demonstrated that both children and adults experience decreased cortisol levels during HAIs with dogs [21,22]. In other studies, cortisol measurements have been used to assess the stress-relieving effects of interactions with cats [23,24]. These studies highlight the potential effectiveness of oxytocin and cortisol level monitoring in evaluating the impacts of HHIs, particularly in terms of stress reduction and social bonding. However, only limited studies have specifically examined the endocrine effects of HHIs, indicating a need for further investigation.

To address this need, the current study aimed to investigate the effects of HHIs by measuring hormone levels in both humans and horses. The primary objective was to assess the impact of interactive activities on both humans and horses by measuring changes in oxytocin and cortisol levels. We hypothesized that interactive activities with horses lead to increased oxytocin levels in humans without significantly affecting their cortisol levels. Similarly, we expected that interactive activities with humans would increase oxytocin levels in horses without significantly affecting their cortisol levels. The findings of this study are anticipated to offer valuable insights into the physiological mechanisms underlying the effects of HHIs.

## 2. Materials and Methods

### 2.1. Participants

Six participants (three women and three men), aged 21 to 25 years (mean ± SD: 22.8 ± 1.7 years), took part in this study. None had a history of clinical diagnosis for physical or psychological conditions, including animal-related trauma. They had limited experience with horses and were unfamiliar with the horses used in this study. However, all participants had previous or current experience owning one or two dogs or cats. All participants provided informed consent and agreed to the experimental procedures, including saliva collection.

### 2.2. Animals

Six mares of various breeds—including Thoroughbred, Haflinger, Quarter Horse, and Pony—were used in this study. The mean age of the horses was 9.7 ± 3.4 years (range: 4–14 years). All mares were in the anestrous phase and had not experienced pregnancy, parturition, or weaning in the year preceding the experiment. They were clinically healthy and had not received any medication during the study. The mares primarily participated in horse training classes and were regularly exercised in the paddock. To minimize the potential influence of training on our results, the experiment was conducted during periods when the horses were not engaged in training sessions. Additionally, no exercise was provided before each activity to prevent confounding effects. Each horse was housed individually in a stall, fed hay three times daily, and provided with concentrate feed twice a day, with water available ad libitum. All horses were managed at the Domestic Animal Research Facility of Kyungpook National University in Sangju, Republic of Korea.

### 2.3. Experimental Procedures

The study was conducted in October, with an average temperature of 18.7 °C and an average humidity of 86.3% during the experimental sessions. Each participant was paired with a specific horse for all activities. The experiment followed a crossover design and was conducted over three consecutive days, with all participants completing a different activity each day. The three 15 min activities were as follows: (1) resting separately (resting), (2) standing with the horses and gazing at them without physical contact (standing), and (3) gently rubbing the horse’s neck and withers (rubbing). Following each activity, the participants and horses were separated for 15 min to allow for a standardized recovery period. The standing and rubbing activities took place in the wash rack adjacent to the stalls. To minimize the impact of circadian rhythms on hormone secretion, all activities were conducted at the same time each morning.

### 2.4. Saliva Samplings

Saliva sample collection was conducted with slight modifications to previously reported methods [25]. Participants were instructed to avoid eating, drinking, or smoking for at least 30 min before saliva collection. Saliva samples were collected at three time points: T0 (baseline, before the activity), T1 (at the end of the activity, 15 min after the start), and T2 (15 min after T1). Approximately 1 mL of saliva was collected from each participant using Salivette tubes (Sarstedt, Nümbrecht–Rommelsdorf, Germany) containing cotton rolls. The cotton rolls were placed under the participant’s tongue for about 1 min to ensure sufficient saliva collection. After collection, the samples were maintained at 4 °C in an icebox until centrifugation. The samples were then centrifuged at 1000× *g* at 4 °C for 2 min to separate the saliva from the cotton rolls. The supernatant was subsequently stored at −80 °C in a freezer until analysis.

### 2.5. Blood Sampling

A catheter (14 gauge × 5.25 inches; 1411, Mila International, Hebron, KY, USA) was inserted into the jugular vein of each horse the day before the designated experimental period for blood sampling. Approximately 10 mL of blood was collected at the same time points as saliva collection. Blood was collected using a syringe inserted into the catheter and subsequently transferred into ethylenediaminetetraacetic acid tubes (BD Vacutainer, Becton Drive, Franklin Lakes, NJ, USA). The collected blood samples were stored in an icebox at 4 °C until centrifugation, which was performed at 1500× *g* at 25 °C for 10 min. The plasma was subsequently stored at −80 °C until assay.

### 2.6. Salivary Oxytocin Enzyme-Linked Immunosorbent Assay (ELISA)

Salivary oxytocin levels were measured using an oxytocin ELISA kit (ADI-900-153A-0001; Enzo Life Sciences, New York, NY, USA), following the manufacturer’s instructions with slight modifications based on a similar study [26]. The kit featured a sensitivity of 15.0 pg/mL. Oxytocin levels were measured at a wavelength of 405 nm using a microplate reader (Tecan, Männedorf, Switzerland). The collected data were analyzed using four-parameter logistic (4PL) curve-fitting software (MyAssays Ltd., Sussex, UK). Notably, the interassay and intraassay coefficients of variation (CVs) were 17.0% and 9.7%, respectively.

### 2.7. Salivary Cortisol ELISA

Salivary cortisol levels were measured using a cortisol ELISA kit (ADI-901-071; Enzo Life Sciences), following the manufacturer’s instructions and with reference to the study by Allen and colleagues [27]. The sensitivity of the kit was 56.72 pg/mL. A steroid displacement reagent was added to the salivary cortisol samples at a 1:100 dilution ratio, followed by further dilution with a sample diluent at a 1:4 ratio. Each sample was analyzed in duplicate. Cortisol levels were measured at a wavelength of 405 nm using a microplate reader (Tecan), and the collected data were analyzed using 4PL curve-fitting software (MyAssays Ltd.). The interassay and intraassay CVs were 15.6% and 9.5%, respectively.

### 2.8. Plasma Oxytocin ELISA

Plasma oxytocin levels were measured using a horse oxytocin ELISA kit (MBS033475; MyBioSource, San Diego, CA, USA) with a sensitivity of 1.0 pg/mL, following the manufacturer’s instructions. Undiluted plasma samples were analyzed in duplicate at a wavelength of 450 nm using a microplate reader (Tecan). The interassay and intraassay CVs were 12.9% and 5.8%, respectively.

### 2.9. Plasma Cortisol ELISA

Plasma cortisol levels were measured using the same cortisol ELISA kit (ADI-901-071; Enzo Life Sciences) as used for salivary cortisol analysis. A steroid displacement reagent was added to the plasma samples at a 1:100 dilution ratio, followed by further dilution with a sample diluent at a 1:8 ratio. The interassay and intraassay CVs were 13.7% and 6.2%, respectively.

### 2.10. Statistical Analysis

The effect of each activity on hormonal levels was evaluated using a mixed general linear model in SAS software (version 9.4; SAS Institute, Cary, NC, USA). Post hoc comparisons of time-dependent hormonal changes for each activity were conducted using the least squares means method. Differences in baseline hormonal levels between women and men were analyzed using independent *t*-tests in SPSS software (version 26; IBM, Armonk, NY, USA). Hormonal levels were presented as the mean ± standard deviation. A *p*-value of <0.05 was considered statistically significant for all analyses.

## 3. Results

### 3.1. Salivary Oxytocin Levels in Humans

We measured salivary oxytocin levels in the male and female participants at three time points: before the activities (T0) and after the activities (T1 and T2). Oxytocin levels remained consistent across the three time points during the resting (control) activity (Table 1). Following the standing activity, oxytocin levels tended to increase at T2 compared to T1 across all participants (*p* = 0.052). However, when analyzed by sex, this trend was observed only in women (*p* = 0.084), with no corresponding pattern in men. Although these increases were not statistically significant in women or across all participants, they suggest a potential trend. No significant differences in oxytocin levels were observed during the rubbing activity for either sex. The total average baseline oxytocin levels across the three activities did not differ significantly between women (64.4 ± 13.6 ng/mL) and men (50.4 ± 3.2 ng/mL).

### 3.2. Salivary Cortisol Levels in Humans

We assessed salivary cortisol levels before and after each activity and analyzed differences between sexes. No significant changes in cortisol levels were observed at any time point during the resting (control) activity (Table 2). Similarly, cortisol levels did not change significantly at any time point during the standing or rubbing activities. The total average baseline cortisol levels across the three activities were significantly higher in women (4.2 ± 1.6 ng/mL) than in men (2.2 ± 0.8 ng/mL) (*p* = 0.005).

### 3.3. Plasma Oxytocin Levels in Horses

Plasma oxytocin levels in horses were measured before and after each activity. Oxytocin levels did not change significantly at any time point during the resting (control) activity (Table 3). During the standing activity, oxytocin levels at T2 significantly surpassed those at T0 (*p* = 0.040). Similarly, during the rubbing activity, oxytocin levels at T2 were significantly higher than those at T0 (*p* = 0.030).

### 3.4. Plasma Cortisol Levels in Horses

We measured plasma cortisol levels in horses before and after each activity. Notably, cortisol levels during the resting activity did not differ significantly across the time points (Table 4). Similarly, cortisol levels did not differ significantly during the standing or rubbing activities.

## 4. Discussion

In this study, we monitored the physiological responses of both humans and horses engaged in HHIs to assess their impact. The study aimed to contribute to a broader understanding of the physiological mechanisms underlying HHIs.

Specifically, we examined changes in oxytocin levels in human saliva after standing with horses and gazing at them. Similar studies have reported that merely observing animals can lead to an increase in salivary oxytocin levels [28]. In our study, oxytocin levels exhibited a slight increasing trend in women; however, this difference was not statistically significant. No similar trend was observed in men. While our findings suggest the potential for sex-based differences in the physiological effects of HHIs, conclusions cannot be drawn based solely on observed trends and should be interpreted with caution. A previous study reported significant differences in hormonal responses between women and men following HAIs [14]. Their findings indicated that interactions with a dog increased oxytocin levels in women but decreased them in men. Additionally, other studies have highlighted sex-based differences in emotional status and competencies following HAIs [29], suggesting that the effects of HHIs may vary by sex. However, given that our study did not identify a statistically significant difference, comparisons with previous research should be made with caution, and further investigation is warranted to clarify the underlying mechanisms.

In this study, no change in oxytocin levels was observed in either men or women following the rubbing activity, suggesting that this interaction may be influenced by additional factors. One potential factor influencing HAI outcomes is prior experience with specific animals [30]. Hama and colleagues found that mood scores during horse-stroking activities significantly differed between individuals with and without prior experience with horses [31]. Their study revealed that individuals without prior experience handling horses reported lower positive mood scores during stroking activities. Similarly, in studies involving dogs, one study reported that participants who had lived with four or more dogs experienced an increase in oxytocin levels after interacting with unfamiliar dogs [32]. Odendaal also found that oxytocin levels increased in humans interacting with familiar dogs but not in those engaging with unfamiliar ones [33]. These findings indicate that both prior experience and familiarity with the animals involved may shape emotional and physiological responses during interactions. The participants in our study were familiar with companion animals, such as dogs and cats, but had little to no prior experience with horses. Whether prior experience with other companion animals influences the effects of interactions with horses remains unclear. Payne and colleagues suggested that the human–horse bond may differ from the human–dog bond due to the distinct roles these animals play in human society [34]. Future studies should explore the impact of prior experience and familiarity with horses, as well as the potential influence of experience with other companion animals, on physiological and emotional responses to human–horse interactions.

We measured cortisol levels during the interactive activities to assess stress responses in humans. The results revealed no significant changes in cortisol levels during either the standing or rubbing activities. Previous studies have reported that activities involving horses do not increase cortisol levels in children [35] or veterans [36] with psychological conditions. These findings suggest that interactive activities with horses, when not involving strenuous exercise, are unlikely to elevate cortisol levels in humans. Additionally, interactions with other companion animals, including dogs and pet goats, have been reported to positively influence the parasympathetic nervous system and cardiovascular function, both of which are physiological indicators associated with stress reduction [37]. Similarly, previous studies have shown that interactions with dogs and cats can have beneficial effects on participants’ anxiety and irritability, both of which are linked to stress [2]. Overall, our findings are consistent with previous research indicating that animal-assisted activities do not induce stress and may instead have a positive impact on stress reduction. These results underscore the potential of interactive activities with animals, particularly horses, as an effective approach for promoting relaxation and overall well-being. Future studies should further investigate the physiological mechanisms underlying these effects and evaluate their long-term benefits across diverse populations.

Several factors may influence the physiological outcomes of HHIs in humans, with interaction duration being a critical factor. This includes both the length of individual sessions and the overall duration of the program. Beetz and colleagues reviewed the timing of oxytocin increases during HAIs [30]. Their analysis of studies on human oxytocin responses following interactions with dogs found that oxytocin levels increased after interactions lasting between 3 and 30 min, depending on study design and experimental conditions. Similarly, Hoagwood and colleagues investigated the effects of therapeutic riding on youth with anxiety [38]. They found that oxytocin levels increased and cortisol levels decreased only after seven weeks of participation, while no significant hormonal changes were observed before this period. This suggests that extending the duration of interactions may need to be considered to elicit measurable hormonal responses. These findings underscore the importance of interaction duration in HHIs and emphasize the need for future research to further examine its impact on physiological outcomes. Furthermore, participants’ prior experience with horses may influence the stress-inducing effects of HHIs. Given their large and powerful nature, horses may evoke fear in some individuals [39]. According to a previous study, the cortisol levels of experienced horse riders significantly decreased during horse riding sessions; however, no changes were observed in inexperienced riders [40]. These findings highlight the need for further research to investigate the differences in physiological responses between individuals with and without horse handling experience. Additionally, Barker and Wolen suggested that the effects of HAIs may be influenced not only by prior experience with animals but also by factors such as the type of interaction (individual vs. group activities) and human attitudes toward animals [37]. Therefore, future research should consider these variables when evaluating the effects of HAIs to ensure more comprehensive and reliable conclusions.

In horses, we assessed plasma oxytocin levels after each interactive activity with humans. Our results revealed a significant increase in oxytocin levels relative to the baseline. Similar findings have been reported in dogs. MacLean and colleagues observed an increase in oxytocin levels in both plasma and saliva samples after 10 min of interaction with humans [41]. Other studies have also reported that oxytocin levels in pet dogs increase through interactions with their owners [42,43]. Payne and colleagues suggested that, as in dogs, affiliative interactions such as petting and scratching contribute to a positive affective state in horses [34,44]. Thus, findings from studies on dogs may provide a useful reference for interpreting the results observed in horses. In contrast to our findings, Malinowski and colleagues did not observe any changes in plasma oxytocin levels at 70 and 90 min following the onset of equine-assisted interventions (EAIs) [6]. Their observations could be attributed to the rapid degradation of oxytocin in the bloodstream. Previous reports indicate that the half-life of oxytocin in mares is approximately 6.8 min [45]. In the study conducted by Malinowski and colleagues, blood collection began 70 min after the program started, likely missing peak oxytocin levels owing to its short half-life in the bloodstream. Overall, these findings suggest that HAIs can stimulate oxytocin secretion in animals, potentially contributing to their well-being.

We measured cortisol levels in horses before and after each interactive activity, observing no significant differences. Consistent with these findings, previous studies on EAIs reported no changes in cortisol levels in horses after riding sessions with children [46,47]. Another study reported that EAIs involving veterans with post-traumatic stress disorder did not elevate cortisol levels in horses [6]. Additionally, in dogs, cortisol levels and stress-associated behaviors did not increase following an animal-assisted activity session [48]. These findings suggest that animal-assisted interventions, including interactive activities, are unlikely to elicit stress responses in animals. Interestingly, Merkies and colleagues reported that the physiological and behavioral responses of horses to humans can be influenced by the humans’ experience with horses [49]. This study suggests that horses’ prior experience with humans may influence the outcomes of HHIs.

Accurately assessing the impact of EAIs on stress responses in horses requires consideration of multiple factors. For instance, it is well established that both the intensity and duration of physical activities can influence cortisol secretion [50,51]. Becker-Birck and colleagues observed that cortisol levels increased in horses during dynamic activities, such as show jumping [52]. Similarly, cortisol levels have been reported to increase in sport horses following intensive training programs [53]. Nagata and colleagues further demonstrated that cortisol responses are significantly associated with the duration of exercise [54]. These findings suggest that the intensity and duration of activities may contribute to stress in horses. Future studies should consider the impact of these physical factors when evaluating cortisol responses in horses following EAIs. Invasive procedures used to monitor cortisol levels can also induce stress in animals. Drude and colleagues demonstrated that single or repeated injections increased corticosterone levels in mice [55]. In our study, catheters were inserted into the horses’ jugular vein one day before the experiments to minimize pain- or fear-induced stress. Future studies should consider noninvasive methods, such as saliva or urine sampling, for measuring cortisol levels in horses [56,57,58]. Careful consideration of these factors in future research will help provide a more accurate understanding of EAIs’ effects on stress in horses.

Despite notable findings in this study, certain limitations should be taken into account when interpreting the results. The limited sample size restricted the strength of our conclusions, particularly in the human data, which were examined based on sex. This underscores the necessity for future studies to incorporate larger populations of both humans and horses to improve the validity and generalizability of these findings. Moreover, there was no data collection to identify potential factors that may have influenced the different oxytocin response patterns observed between female and male participants. One study found that the level of connection to animals varies by sex, suggesting that these differences in perception could influence the outcomes of HHIs [59]. Therefore, future research should more precisely evaluate and compare participants’ perceptions and experiences with animals to better understand their potential influence on physiological responses. Additionally, comparing our results with other stress-related indicators could have strengthened this study’s conclusions. Future research should incorporate additional physiological and behavioral measures, such as heart rate and behavioral observations, to provide a more comprehensive assessment of stress responses.

## 5. Conclusions

In conclusion, our findings demonstrate the potential of HHIs to promote social bonding in both women and horses. Notably, the interactive activities involved in our HHIs did not trigger a stress response in either humans or horses. Overall, this study provides valuable insights into the hormonal changes associated with HHIs.

## Figures and Tables

**Table 1:** Human salivary oxytocin levels over time by activity (pg/mL).

	Resting			Standing			Rubbing		
	T0	T1	T2	T0	T1	T2	T0	T1	T2
Women	55.3 ± 38.6	42.5 ± 27.6	74.9 ± 40.9	73.6 ± 63.7	58.1 ± 41.9	119.8 ± 65.2	64.4 ± 29.5	37.4 ± 10.2	66.5 ± 21.1
Men	51.7 ± 15.9	54.7 ± 15.0	60.0 ± 21.5	49.9 ± 7.0	48.7 ± 10.0	58.3 ± 2.8	49.3 ± 2.2	41.9 ± 8.3	42.1 ± 1.8
Total	53.5 ± 26.5	47.4 ± 21.9	67.4 ± 30.3	64.1 ± 47.0	53.4 ± 27.7	89.1 ± 53.2	56.9 ± 20.5	39.7 ± 8.7	54.3 ± 18.9

**Table 2:** Human salivary cortisol levels over time by activity (ng/mL).

	Resting			Standing			Rubbing		
	T0	T1	T2	T0	T1	T2	T0	T1	T2
Women	4.2 ± 1.3	4.3 ± 1.5	3.1 ± 1.5	3.6 ± 0.7	4.1 ± 2.8	3.6 ± 2.5	4.6 ± 2.4	3.3 ± 0.7	2.9 ± 1.2
Men	2.0 ± 1.2	1.9 ± 0.2	2.0 ± 0.8	2.1 ± 0.3	2.2 ± 1.6	2.2 ± 0.6	2.4 ± 1.0	3.0 ± 1.2	2.4 ± 1.3
Total	2.9 ± 1.6	2.8 ± 1.6	2.5 ± 1.1	2.7 ± 0.9	3.2 ± 2.3	2.9 ± 1.8	3.5 ± 2.1	3.1 ± 0.9	2.6 ± 1.2

**Table 3:** Horse plasma oxytocin levels over time by activity (pg/mL).

	Resting			Standing			Rubbing		
	T0	T1	T2	T0	T1	T2	T0	T1	T2
Oxytocin levels (pg/mL)	18.8 ± 8.6	18.5 ± 7.1	20.4 ± 4.3	14.5 ± 5.0 ^a^	18.3 ± 4.7 ^ab^	22.2 ± 3.8 ^b^	13.6 ± 4.5 ^a^	19.4 ± 3.4 ^ab^	21.7 ± 7.5 ^b^

^a,b^ Different superscript letters denote significant differences (*p* < 0.05).

**Table 4:** Horse plasma cortisol levels over time by activity (ng/mL).

	Resting			Standing			Rubbing		
	T0	T1	T2	T0	T1	T2	T0	T1	T2
Cortisol levels (ng/mL)	7.2 ± 3.3	8.7 ± 3.2	7.9 ± 1.8	5.7 ± 2.5	5.5 ± 1.3	5.6 ± 1.4	6.4 ± 3.2	6.8 ± 2.2	7.4 ± 3.4

## Data Availability

The original contributions presented in this study are included in the article. Further inquiries can be directed to the corresponding author.
